# Curcumin as an indirect methylation inhibitor modulates the effects of *Toxoplasma gondii* on genes involved in male fertility

**DOI:** 10.17179/excli2020-2052

**Published:** 2020-08-24

**Authors:** Jasem Saki, Mohamad Sabaghan, Reza Arjmand, Ali Teimoori, Mohammad Rashno, Ghasem Saki, Saeedeh Shojaee

**Affiliations:** 1 Cellular and Molecular Research Center, Ahvaz Jundishapur University of Medical Sciences, Ahvaz, Iran; 2 Department of Parasitology, Faculty of Medicine, Ahvaz Jundishapur University of Medical Sciences, Ahvaz, Iran; 3 Virology Department, School of Medicine, Ahvaz Jundishapur University of Medical Sciences, Ahvaz, Iran; 4 Department of Immunology, School of Medicine, Ahvaz Jundishapur University of Medical Sciences, Ahvaz, Iran; 5 Physiology Research Center, School of Medicine, Ahvaz Jundishapur University of Medical Sciences, Ahvaz, Iran; 6 Department of Medical Parasitology and Mycology, School of Public Health, Tehran University of Medical Sciences, Tehran, Iran

**Keywords:** Toxoplasma gondii, DNMTs, gene expression, NF-kappaB, DNA methylation

## Abstract

*Toxoplasma gondii* is a common protozoan parasite, which infects warm-blooded mammals, including mice and humans, throughout the world. The negative effects of *T. gondii *infection on the human reproductive system have been documented, especially in females. However, only few studies have examined the effects of *T. gondii *infection on the male reproductive system. Previous research shows that *T. gondii* can induce DNA methylation in some gene promoters, which are key regulators of spermatogenesis. Therefore, this study aimed to evaluate the effects of curcumin on the activity of DNA methyltransferases (DNMTs), as well as selected genes, involved in spermatogenesis in spermatogenic cells. In the spermatogenic cells exposed to *T. gondii*, there was a significant increase in DNMT1 and DNMT3A gene expression and a significant reduction in HSPA1A, MTHR, and DAZL gene expression, compared to the controls. The present results showed that curcumin could regulate changes in *T. gondii*-mediated gene expression. The effect of *T. gondii* on DNMT activity was also investigated in this study. A 40 % increase in DNMT activity was observed due to *T. gondii* infection. However, DNMT activity was restored by treatment with 20 μM curcumin for eight hours. The results revealed that *T. gondii* increases the NF-κB activity, compared to the control group. The increase in NF-κB activity, induced by *T. gondii*, was inhibited by curcumin. In conclusion, *T. gondii*, by increasing DNMT expression and activity, leads to an increase in NF-κB activity in cells. On the other hand, curcumin reduced DNA methylation, induced by *T. gondii,* owing to its NF-κB-inhibiting properties. Therefore, curcumin, as a hypomethylating agent, can be potentially used to alleviate the negative effects of *T. gondii* on the male reproductive system.

## Introduction

*Toxoplasma gondii *(*T. gondii*), as a ubiquitous intracellular protozoan parasite, can cause toxoplasmosis in humans, as well as other warm-blooded mammals. It is estimated that more than one billion people are infected with* T. gondii* around the world (Ahmadpour et al., 2014[2]; Dubey and Jones, 2008[12]). Evidence shows that *T. gondii* cannot cause serious diseases in most human adults. However, if a pregnant woman is infected with this parasite for the first time, it may lead to severe complications for the fetus. 

Besides, a significant relationship has been reported between the quality of sperms and *Toxoplasma *infection (Dubey and Jones, 2008[12]; Zhou et al., 2003[52]). According to some previous studies, fertility decreases in rats following infection with *T. gondii*. Besides,* T. gondii* infection reduces the epididymal weight, sperm concentration, and sperm motility and promotes abnormal sperm morphology. Moreover, a significant relationship has been reported between toxoplasmosis and increased sperm apoptosis, particularly of diploid spermatocytes (Terpsidis et al., 2009[42]; Yang et al., 2006[50]). It is known that male infertility is significantly associated with different factors, including environmental factors, genetic factors, and infections (Cui et al., 2016[10]). However, the mechanisms underlying male infertility are unknown in 70 % of cases, even if the cause of infertility is known. 

The negative effects of* T. gondii* infection on the reproductive system of humans have been described in the literature, particularly in females (Colosi et al., 2015[9]). However, only a limited number of studies have examined the effects of this infection on the male reproductive system. There is also no information available on how* T. gondii* changes the reproductive parameters (Dvorakova-Hortova et al., 2014[13]; Eslamirad et al., 2013[16]). Nevertheless, pathogens can regulate the host cell immunological reaction through manipulation of epigenetic processes, associated with chronic infections (Paschos and Allday, 2010[32]). Besides bacteria and viruses, microparasites (e.g., protozoa), macroparasites, and environmental factors can cause pathological alterations in the infected host cell epigenome. They can hypo- or hyper-methylate particular gene promoters, including those responsible for continuous spermatogenesis (Rajender et al., 2011[34]; Trasler, 2009[44]). Nevertheless, it is possible to reverse or avoid specific pathoepigenetic changes if they are well understood; therefore, such information can be of therapeutic importance (Minarovits, 2009[27]). 

The DNA methylation process is dependent on DNA methyltransferases (DNMTs), as a family of five members, including DNMT3a, DNMT3b, and DNMT1 with catalytic activities. DNMT1 is defined as the maintenance DNA methyltransferase, whereas DNMT3B and DNMT3A function as de novo methyltransferases (Minarovits, 2009[27]). Methylation in mammals almost exclusively occurs at short DNA sequences, called CpG islands. These islands consist of about 5-10 CpGs per 100 bp in length. Up to 80 % of CpG islands are located in non-coding DNA regions, which majorly contribute to the global methylation condition (Auclair and Weber, 2012[4]). 

Curcumin is a primary yellow pigment, extracted from turmeric. It is commonly used as a dietary additive to enhance the color and flavor of foods. It has been traditionally consumed as a treatment for skin wounds, inflammation, cough, and some tumors (Akram et al., 2010[3]). According to recent studies, it may exert its effects through epigenetic modifications, such as DNMTs, histone deacetylase (HDAC), and histone acetyltransferase (HAT). This compound is recognized as a potential DNMT inhibitor, which results in the hypomethylation of different genes (Link et al., 2013[24]; Reuter et al., 2011[36]).

Aberrant DNA methylation in male germ cells causes abnormal testicular histology and alters spermatogenesis in humans and mice (Terpsidis et al., 2009[42]; Wu et al., 2010[47]). According to previous studies, *T. gondii* can trigger DNA methylation in some gene promotors, as major regulators of spermatogenesis. Therefore, the current study aimed to investigate the effects of curcumin on the activity of DNMTs, as well as selected genes associated with spermatogenesis in spermatogenic cells. 

## Materials and Methods

### Ethical statement

In this study, the animals were kept according to the International Guidelines on the Use of Laboratory Animals, as well as the ethical guidelines of the Animal Care Committee of Jundishapur University of Medical Sciences, Ahvaz, Iran. Also, the Ethics Committee of Jundishapur University of Medical Sciences approved the procedures in this study (IR.AJUMS.REC.1395.117). The animals’ health was verified, using the health surveillance program tests, according to the guidelines by the Federation of European Laboratory Animal Science Associations (FELASA). The researchers made their best efforts to minimize the animals’ suffering.

### Experimental design, animals, and tachyzoites

Three BALB/c mice, aged 6-8 weeks (weight: 20-22 g), were used in this study. The laboratory animal breeding unit of Razi Vaccine and Serum Research Institute of Iran provided the animals. The mice were acclimatized to the laboratory conditions for seven days before the experimental treatments. Standard plastic cages were used for housing the animals, which were kept under controlled laboratory conditions (temperature, 22-23 °C; humidity, 55 %) in a 12:12 h light-dark cycle. The mice were fed a normal commercial chow and water ad libitum. 

The virulent RH strains of *T. gondii* were grown and kept by routine intraperitoneal (IP) injection in BALB/c mice, provided by the Parasitology and Mycology Department of Tehran University of Medical Sciences, Tehran, Iran. To expose spermatogenic cells to fresh tachyzoites, tachyzoites (2×10^2^) were intraperitoneally injected to mice. The animals were then placed in separate cages for one week until signs of general weakness and peritonitis emerged. Upon observation of the signs, they were euthanized with intramuscular injection of xylazine 5 % (10 mg/kg) and ketamine 10 % (60 mg/kg), according to the ethical standards of the Ethics Committee of Jundishapur University of Medical Sciences, Ahvaz, Iran. Samples containing fresh tachyzoites were collected from the peritoneal fluid under sterile conditions. After the samples were collected from the infected mice, they were rinsed twice at 1000×g for 15 minutes in sterile phosphate-buffered saline (PBS; pH=7.2). Finally, tachyzoites (2×10^2^) were injected into other mice.

### Cell viability assessment

To investigate the effect of curcumin on cell viability, seeding was carried out at a density of 2.0×10^4^ cells/well in a 96-well plate. Next, the cells were subjected to curcumin for eight hours with increasing concentrations (0.1-50 µM). They were subjected to 20 µM curcumin for 1-24 hours to determine the time-dependent impact of curcumin treatment on the viability of cells. MTT (1.6 mg/mL) was also added to the cells in each well. Afterward, incubation was performed for four hours at 37 °C. Following the solution removal, the cells were re-suspended in 100 µL of dimethyl sulfoxide (DMSO). The optical density was read at 540 nm, and background was subtracted at 670 nm. The viability of cells (%) was determined as follows:

Cell viability= (OD_treatment_/OD_control_) × 100

### Cell culture and treatment of T. gondii infection

The GC-1 spg cell line, provided by the National Cell Bank of Pasteur Institute of Iran, was used in this study. After preparing the cell line and confirming the lack of contamination in the medium, the viability of cells was studied by trypan blue 0.4 %. Next, the cells were counted manually, using a hemocytometer. The Dulbecco’s Modified Eagle Medium (DMEM; Gibco, USA), supplemented with 100 U/mL of penicillin, 100 μg/mL of streptomycin, and 10 % (v/v) fetal bovine serum, was used to culture the cells. Next, the cells were incubated in 5 % CO_2_ at 37 °C. Tachyzoites were pelleted for ten minutes by centrifugation at 800×g and re-suspended in lysis buffer (150 mM NH_4_Cl and 0.1 M Tris–HCl, pH=7.4) at ambient temperature for ten minutes. *T. gondii* tachyzoites infected the target cells at a multiplicity of infection (MOI) of 1:1 in 100 μL of DMEM complete medium for 18 hours. Afterward, the cells were subjected to 25 mM curcumin for six hours and treated for further analyses. Finally, the effects of curcumin on DNMT activity, p65 component of NF-κB, and expression of fertility genes were investigated in GC-1 spg cells.

### Quantitative real-time polymerase chain reaction (qRT-PCR) assay

Trizol^®^ Reagent (Invitrogen, Carlsbad, CA, USA) was used for extracting total cellular RNA. RNA was reverse-transcribed into single-stranded cDNA, using the SuperScript III Reverse Transcriptase, according to the manufacturer’s protocol. The expression level of mRNA was determined by TaqMan Gene Expression Assay for qRT-PCR, using a 7300 real-time PCR system. The cycle threshold (Ct) values of the samples were measured to determine the number of cell equivalents in the experimental samples. The cycling conditions and design of primer sequences were performed as previously described, and data were normalized relative to GAPDH expression (Table 1(Tab. 1)). 

### Evaluation of NF-κB p65 activity by ELISA assay 

To investigate the effect of curcumin on NF-κB p65 activity, incubation was performed for 48 hours, and then, nuclear extracts were obtained, using a nuclear extraction kit (ab113474, Abcam, UK), according to the manufacturer’s instructions. A sensitive ELISA assay was used for determining the nuclear p65 concentration. In brief, a specific double-stranded DNA (dsDNA) sequence, with the NF-κB p65 response element, was immobilized on the bottom of the wells in a 96-well plate. Generally, the p65 component in the nuclear extract particularly binds to the NF-κB p65 response element. The addition of a specific primary antibody against NF-κB p65 helps detect this component. Next, a secondary antibody, conjugated to horseradish peroxidase (HRP), was added. Finally, a spectrophotometer was employed for reading absorbance at 450 nm (Malaponte et al., 2015[26]; Saha et al., 2017[39]).

### Quantification of DNMT activity

A colorimetric DNMT activity quantification kit was used to assess the DNMT activity. This kit is appropriate for the assessment of total DNMT activity, according to the manufacturer’s instructions. In brief, 50 μL/well of the reaction solution was used for diluting 7.5 ng of nuclear extracts. The 96-well plate with positive and blank controls was covered, and incubation was performed for two hours at 37 °C. After removing the reaction solution, the wells were rinsed three times with 150 μL of wash buffer, and then, 50 μL/well of the diluted capture antibody was added. An aluminum foil was used for covering the plate, and incubation was performed for one hour at ambient temperature. After the capture antibody was removed, the wells were rinsed three times with 150 μL of wash buffer. Next, 50 μL/well of the diluted detection antibody was added. An aluminum foil was used for covering the plate, followed by incubation for 30 minutes at ambient temperature. 

After removing the detection antibody, the wells were rinsed four times with 150 μL of wash buffer, and then, 50 μL/well of the enhancer solution was added. An aluminum foil was used for covering the plate, followed by incubation for 30 minutes at ambient temperature. After removing the enhancer solution, the wells were rinsed five times with 150 μL of wash buffer, and then, 100 μL/well of the developer solution was added. An aluminum foil was used for covering the plate, followed by incubation for ten minutes at ambient temperature; there was no direct exposure to light. After the color of the positive control turned to medium blue, 100 μL/well of stop solution was added to terminate the enzyme reaction. A microplate reader was used for reading absorbance at 450 nm during 2-10 minutes. The selected reference wavelength was 655 nm. The DNMT activity is expressed as control percentage.

### Statistical analysis

GraphPad Prism 6 was used for data analysis. Data are reported as mean±SD. To compare two independent groups, student t-test was used, and to compare multiple samples, ANOVA test was performed. P-value below 0.05 was considered statistically significant.

## Results

### Effect of curcumin on GC-1 spg cells 

**Figure 1:**
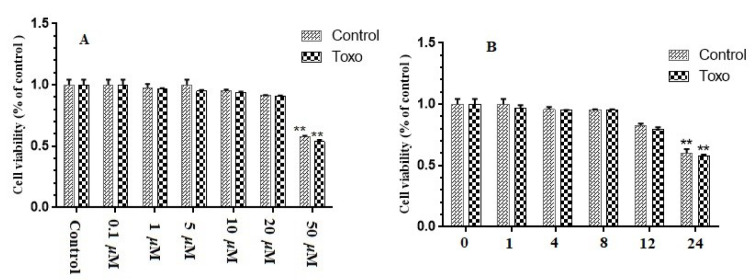
Effect of curcumin on viability of GC-1 spg cells. (A) Thiazolyl blue tetrazolium bromide (MTT) assay run on GC-1 spg cells, which was kept in infection or normal conditions. Then, the cells were subjected to rising concentrations (0.1–50 µM) of curcumin for eight hours. (B) MTT assay run on GC-1 spg cells, which were kept in infection or normal conditions. Then, the cells were subjected to 20 µM curcumin for one hour to one day. ^∗^ p < 0.05; ^∗∗^ p < 0.01.

In this study, cytotoxicity of curcumin in GC-1 spg cells, cultured in a 96-well plate, was determined. The cells were subjected to curcumin (0.1-50 μM) with varying concentrations for eight hours or 20 μM curcumin for different exposure periods. No significant cytotoxic effect was reported due to treatment with 0.1-20 μM curcumin, while treatment with 50 μM curcumin for eight hours led to a 43 % reduction in cell viability (P<0.01). Besides, treatment with 20 μM curcumin for up to 12 hours did not exert any significant effects on the viability of cells. Nevertheless, viability of cells decreased by 24 % (P=0.183) and 36 % (P<0.01) of non-treated controls after exposure to 20 μM curcumin for 12 and 24 hours, respectively. Accordingly, treatment with 20 μM curcumin for eight hours was selected for further analyses to prevent possible cytotoxicity (Figure 1(Fig. 1)).

### Dnmts gene expression 

**Figure 2:**
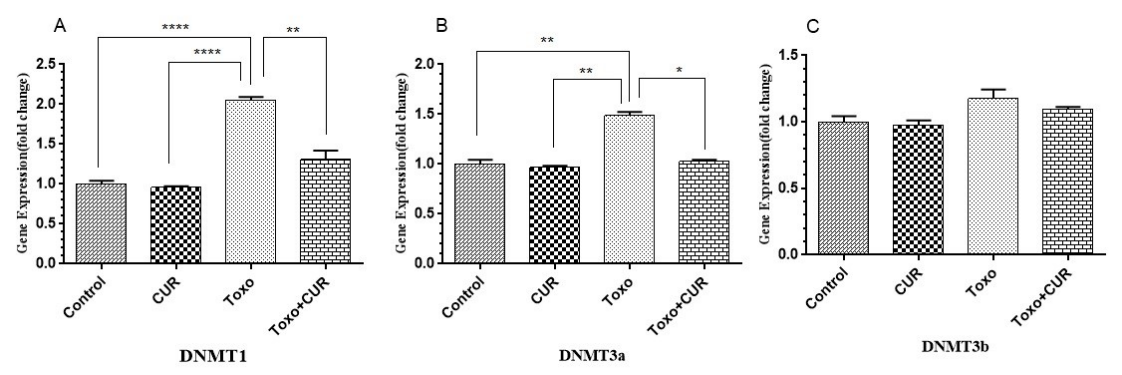
DNMT1, 3a and 3b gene expression in GC-1 spg cell following treatment with CUR; Curcumin, Toxo; *Toxoplasma gondii*, Toxo+CUR; Curcumin +*Toxoplasma gondii* ***P < 0.00001, ***P < 0.0001, **P < 0.0001, *P < 0.05

The spermatogenic cells, exposed to *T. gondii*, showed a significant increase in the level of DNMT1 (P<0.00001) and DNMT3A (P<0.001) gene expression, compared to the controls. The results showed that curcumin could inhibit the *T. gondii*-mediated increase in DNMT1 and DNMT3A gene expression. However, no significant difference in the expression level of DNMT3B gene was reported (Figure 2(Fig. 2)).

### Male related fertility genes expression 

**Figure 3:**
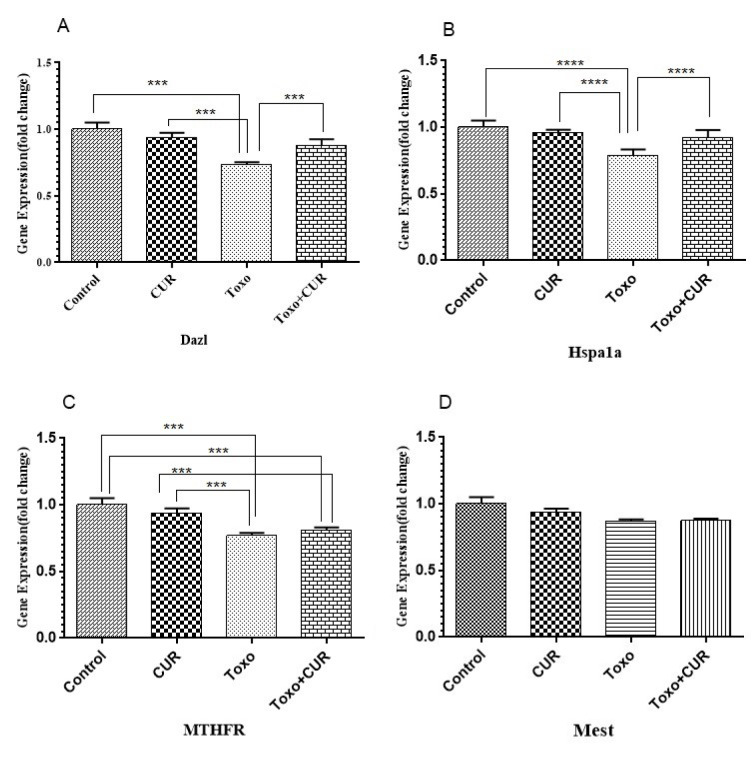
*Dazl, Hspa1a, MTHFR *and* Mest* gene expression in GC-1 spg cell following treatment with CUR; Curcumin, Toxo; *Toxoplasma gondii*, Toxo+CUR; Curcumin +*Toxoplasma gondii*. ***P < 0.00001, ***P < 0.0001, **P < 0.0001, *P < 0.05.

Comparison with the control group showed that the expression of HSPA1A, MTHFR, and DAZL genes reduced significantly in *T. gondii*-exposed mice (P<0.001). On the other hand, the *T. gondii*-mediated change in HSPA1A and DAZL gene expression was significantly diminished by curcumin (P<0.05 and P<0.01, respectively). The results indicated that MTHFR gene expression did not change significantly in the curcumin-treated control group. *T. gondii* did not cause a significant change in MEST gene expression (Figure 3(Fig. 3)). 

### Curcumin restores levels of DNMT and NF-κB activity 

**Figure 4:**
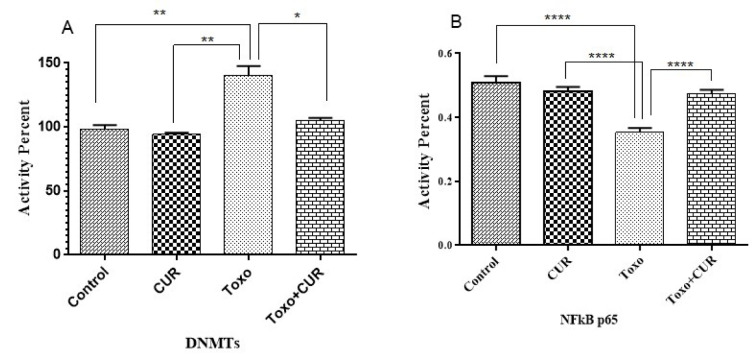
p65-NF-κB and DNMT activity in cells following treatment with CUR; Curcumin, Toxo; *Toxoplasma gondii*, Toxo+CUR; Curcumin +*Toxoplasma gondii*. ***P < 0.00001, ***P < 0.0001, **P < 0.0001, *P < 0.05

The effect of *T. gondii* on DNMT activity was also examined in this study. A 40 % increase was observed in DNMT activity due to *T. gondii* infection (P<0.05). However, DNMT activity was restored by treatment with 20 μM curcumin for eight hours. To investigate the effect of curcumin on NF-κB p65 activity in cells, infected with *T. gondii*, the DNA binding activity of NF-κB was analyzed by ELISA assay. The results revealed that *T. gondii* increased the NF-κB activity, compared to the control group. However, the increase in NF-κB activity, induced by *T. gondii*, was inhibited by curcumin (Figure 4(Fig. 4)).

## Discussion

Various factors, including environmental factors, genetic factors, and infections, are involved in male infertility (Cui et al., 2016[10]). The mechanisms underlying male infertility are unknown in 70 % of cases, even when the cause of infertility is determined. The negative effects of *T. gondii* infection on the human reproductive system have been described in the literature, particularly in females (Colosi et al., 2015[9]). However, only a limited number of studies have examined the effects of *T. gondii* infection on the male reproductive system (Abdoli et al., 2012[1]; Dalimi and Abdoli, 2013[11]).

Epigenetics is the study of genomic structural variations, which affect the expression of genes, without changing the basic nucleotide sequence (Verma et al., 2014[45]; Xu and Song, 2014[48]). DNA methylation is involved in many biological functions, including spermatozoa development, early embryonic development, and repression of endogenous retrotransposons. Moreover, the effects of DNA methylation on gene expression have been reported in the literature (Cheng et al., 2014[8]; Trasler, 2009[44]). Evidence suggests that dysregulation of DNA methylation is associated with different human disorders, as it increases the risk of fertilization failure, embryogenesis dysfunction, congenital abnormalities, perinatal mortality, low birth weight, and preterm birth (Chen et al., 2015[7]; Griseri et al., 2016[17]; Wang et al., 2014[46]). 

According to previous research, *T. gondii* has the potential to cause pathological changes via specific epigenetic alterations, such as abnormal DNA methylation of certain genes, which are important in spermatogenesis (Zhu et al., 2011[53]). However, the mechanism underlying the impact of *T. gondii* on DNA methylation remains unknown. In the current study, the effects of *T. gondii* on DNMT activity and gene expression were evaluated. The results indicated that *T. gondii* infection causes an increase in the expression of *DNMT1* and *DNMT3A* genes in spermatogenic cells, compared to the control group. Moreover, the increased DNMT activity was significantly higher in the *T. gondii* group, compared to the control group. Based on these findings, *T. gondii* can affect DNA methylation by altering DNMT activity and expression. 

In the current study, the increase in DNMT activity and expression, caused by *T. gondii,* was prevented by curcumin treatment in spermatogenic cells. Based on the findings, the *T. gondii*-mediated increase in NF-κB activity was reduced by curcumin in spermatogenic cells. Generally, intracellular pathogens have provided different strategies for evading destruction by the host immune system. The induction of apoptosis is a defense mechanism against infected cells for counteracting the invading pathogen. Infection of various human and mouse cell lines with *T. gondii *prevents apoptosis, caused by different stimuli, such as Fas-dependent cytotoxicity, TNF-α treatment, actinomycin D, growth factor deprivation, and ultraviolet irradiation. 

NF-κB majorly contributes to the regulation of anti-apoptotic protein expression (Cahir-McFarland et al., 2000[6]). It leads to the expression of genes with products, which can prevent apoptosis, including Bcl-2 members (BFL-1 and Bcl-xL), TNF-receptor-associated factors (TRAF1 and TRAF2), and cellular inhibitors of apoptosis proteins (c-IAP) (Karin and Lin, 2002[20]). However, there are contradictory results regarding the potential of *T. gondii* in NF-κB activation (Shapira et al., 2002[41]). It is known that NF-κB regulates the expression of DNMTs (Lu and Stark, 2015[25]; O'Gorman et al., 2010[30]). Yu et al. reported that curcumin leads to the reduction of *DNMT1* mRNA and protein expression, probably by inhibiting the expression of positive regulators (DNMT1), including p65 and Sp1 subunits of NF-κB, and/or alteration of their potential to bind to the promoter region of *DNMT1 *gene (Yu et al., 2013[51]). 

Generally, curcumin can have several functions, as it can act as a chemical inhibitor or a transcriptional modulator of DNMT1. In this regard, Tong et al. showed that curcumin led to the inhibition of NF-κB through AMPK activation (Tong et al., 2016[43]). A significant decrease in the expression of *HSPA1A, MTHFR,* and *DAZL *genes was observed in *T. gondii*-exposed spermatogenic cells, compared to the controls. According to the present findings, curcumin can inhibit the *T. gondii*-mediated reduction of *HSPA1A* and *DAZL *genes. However, no changes were observed in *MTHFR *gene expression*.*

DNA hypermethylation is related to gene silencing, whereas DNA hypomethylation contributes to gene expression. Promoters of developmental genes are considerably hypomethylated in sperm cells. As shown by an earlier gene ontology analysis, hypomethylation in mature sperm cells results in the promotion of signaling and transcription, bound by self-renewal of mesh transcription factors in human embryonic stem cells, such as SOX2, OCT4, KLF4, NANOG, and FOXD3 (La Salle and Trasler, 2006[22]; Omisanjo et al., 2007[31]; Rousseaux et al., 2006[38]). Dvorakova-Hortova et al. detected a significant increase in DNA methylation in a Toxo^+^ group of mice. They provided primary evidence regarding the potential of *T. gondii* in modification of the host epigenomes. However, it is not known how *T. gondii* can manipulate the epigenetic machinery of the host. 

Moreover, Dvorakova-Hortova et al. indicated that methylation of *HSPA1* gene promoters was significantly higher in the Toxo^+^ group, compared to the Toxo^−^ group (Dvorakova-Hortova et al., 2014[13])*.* For the assembly and function of protein complexes involved in energy production, post-meiotic germ cells require epigenetically regulated *HSPA1* genes, which are responsible for coding a testis-specific heat shock cognate protein (HSC70t) (Eddy, 2002[15]). Therefore, the increased *HSPA1* gene methylation may lead to reduced protein expression, which contributes to adjusted sperm motility, as shown in rats and humans with *Toxoplasma *infections (Terpsidis et al., 2009[42]). 

DAZL is a member of the DAZ gene family, which is responsible for encoding RNA-binding proteins, required for the development of germ cells in various organisms (Haston et al., 2009[19]). Evidence suggests that different species require DAZL for the development of germ cells (Eberhart et al., 1996[14]; Lin and Page, 2005[23]). This gene is recognized as a gene cluster with deletions in at least 10 % of men with oligozoospermia or azoospermia (Reijo et al., 1995[35]; Saxena et al., 1996[40]). Therefore, it is considered a suitable alternative for treating male infertility. According to previous studies on patients with oligoasthenoteratozoospermia, there was an increase in methylation defects of *DAZL* promoters. Aberrant DNA methylation of DAZL promoter indicated an epigenetic marker, associated with infertility in males (Navarro-Costa et al., 2010[29]).

One of the major enzymes in the folate metabolic pathway is methylenetetrahydrofolate reductase (MTHFR), which has a major function in establishing balance in the pool of methyl groups during DNA methylation and synthesis. Any alterations in the *MTHFR *gene sequence may regulate spermatogenesis, leading to infertility transmission. There are two possible mechanisms for dysregulation of *MTHFR *gene expression, that is, aberrant promoter methylation and gene mutation (Botezatu et al., 2014[5]; Khazamipour et al., 2009[21]; Rotondo et al., 2013[37]). Hypermethylation of *MTHFR* gene promoter in spermatozoa seems to be associated with idiopathic male infertility (Rotondo et al., 2013[37]).

In the current study, there was no significant difference between the *T. gondii* group and the other groups in terms of *MEST* gene expression. *T. gondii* infection could not affect *MEST *gene expression, compared to the control group. In this regard, Xu et al. indicated that the risk of low sperm motility increased due to the reduction of DNA methylation at certain imprinted loci (GNAS, FAM50B, and MEST) (Xu et al., 2016[49]). Research shows that patterns of DNA methylation in spermatozoa of patients with infertility significantly change at different imprinted loci (MEST, LIT1, SNRPN, PEG3, PLAGL1, IGF2, and H19) (Hammoud et al., 2010[18]; Minor et al., 2011[28]). Besides, a relationship was found between aberrant DNA methylation of *MEST* gene, poor sperm morphology, and decreased progressive sperm motility (Poplinski et al., 2010[33]). In the present study, curcumin downregulated DNMT activity and expression, which led to DNA hypomethylation and gene reactivation in the cells. Therefore, hypomethylating agents (e.g., curcumin) are responsible for demethylation of CpG islands, associated with increased *MTHFR* gene expression. 

In conclusion, according to the present findings, *T. gondii* can increase the NF-κB activity in cells by increasing the DNMT activity and expression. Moreover, curcumin reduces DNA methylation, caused by *T. gondii* due to its NF-κB-inhibiting properties. Therefore, curcumin, as a hypomethylating agent, has the potential to reduce the negative effects of *T. gondii* on the male reproductive system. 

## Acknowledgements

This study originates from the thesis of Mr. Mohamad Sabaghan, a Ph.D. student of medical parasitology, granted by the Ahvaz Jundishapur University of Medical Science (CMRC-9505).

## Authors’ contributions

JS and MS conceived and designed the study. MS, RA and JS performed the article search and data extraction. JS, AT, MR and SS evaluated the methodological quality of each study. AT, GS and MS analyzed the data and wrote the paper, which was improved by JS by supervising the research. All authors read and approved the final manuscript.

## Conflict of interest

The authors declare that they have no conflict of interest.

## Figures and Tables

**Table 1:**
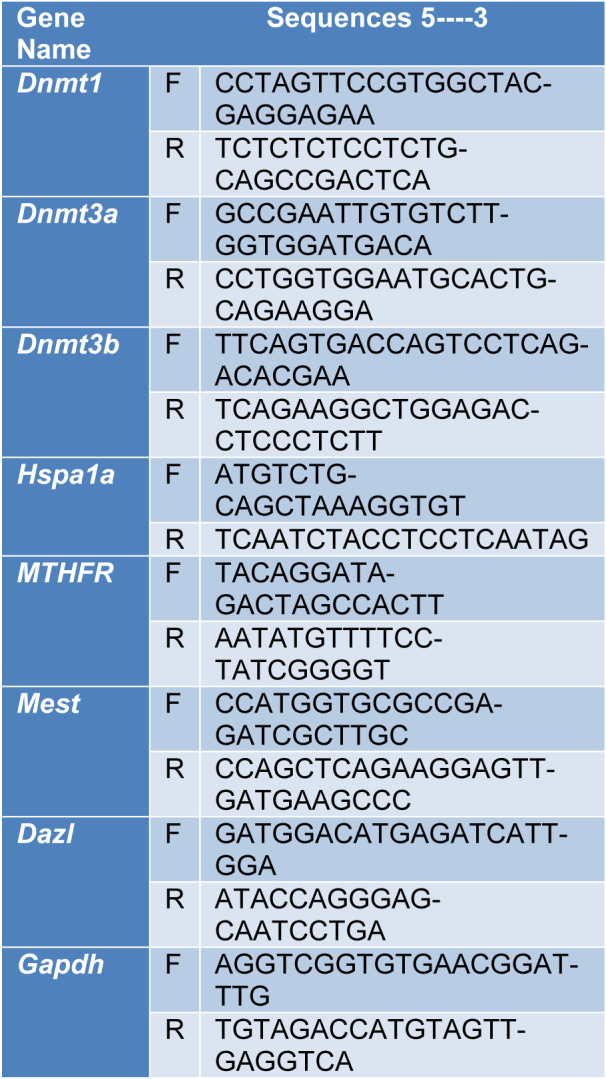
Primer sequences
